# Status Epilepticus After Electrocution Injury in a Dog

**DOI:** 10.1111/vec.70103

**Published:** 2026-03-27

**Authors:** Julia Ortlieb, Sofie Muthmann, Fabienne Blunschi, Adriana Czerwik, Agnieszka Olszewska, Esther Hassdenteufel

**Affiliations:** ^1^ Department of Veterinary Clinical Sciences, Small Animal Clinic Justus‐Liebig‐University Giessen Giessen Germany

**Keywords:** EEG, electrical injury, electrocution, electrocution injury

## Abstract

**Objective:**

To describe a case of status epilepticus secondary to electrocution injury associated with an electric fence.

**Case Summary:**

A 1.5‐year‐old male Kangal Shepherd Dog mix was presented to the emergency service after being entangled in an agricultural electric fence for 1–2 h. On presentation, the dog was recumbent, was unable to stand or walk, and showed severe hypersalivation, panting, and tachycardia. No electrical burn injuries were observed, and the remaining physical and neurologic examination parameters were normal. ECG and thoracic radiographs showed no abnormalities. During further assessment and stabilization of the patient, generalized tonic‐clonic seizures occurred. Initial treatment with antiseizure medications had no effect, and antiseizure therapy was escalated in response to the refractory seizure activity. With escalation of the antiseizure therapy, the dog became unable to sufficiently ventilate, requiring initiation of mechanical ventilation. Given the rapid deterioration and refractory seizure activity, a 24‐h electroencephalogram (EEG) was performed to guide further treatment and provide prognostic information. EEG monitoring revealed physiologic background activity, and the dog was successfully weaned after a total of 30 h of mechanical ventilation. Antiseizure therapy was slowly tapered throughout the remaining hospital stay, and the dog made a full neurologic and clinical recovery and was discharged with oral phenobarbital after 7 days. Four weeks after discontinuation of phenobarbital, the dog experienced a recurrence of seizure activity, and medication was reinitiated.

**New or Unique Information Provided:**

To the authors’ knowledge, this is the first case describing the successful management, including ventilation and EEG monitoring, of a dog with status epilepticus secondary to electrocution injury associated with an electric fence.

**Abbreviations**
AC: alternating current
CRI: constant rate infusion
DC: direct current
EEG: electroencephalogram
PEEP: positive end‐expiratory pressure
RI: reference interval

## 1 Introduction

Electric fences are commonly used to protect and confine livestock by supplying a brief, high‐voltage, low‐current impulse. Such fences are typically considered harmless to people and animals. Electric fences usually consist of a direct current (DC) power source, such as a battery, and an energizer to convert the power into a brief, high‐voltage pulse. Pulse duration is usually between 0.1 and 0.3 ms, with a prolonged interval of 1–2 s between each impulse. When an animal touches the fence during a pulse, it closes the electric circuit. The electrical current flowing through the animal to the ground will be felt as a brief, painful electric shock, resulting in future avoidance of the fence. Due to the brief impulses and the intervals between each pulse, electric fences are generally considered harmless, despite the high voltage. Compared with continuous alternating current (AC), the short pulse flow and DC prevent muscle contractions from occurring, thus allowing animals to step away from the fence unharmed. However, rare reports exist of fatal encounters in people [1] and wildlife [2] after accidentally becoming entrapped in electric fences.

A DC power source can have an unpredictable effect on the central nervous system in animals, ranging from transient behavioral changes to severe epileptic seizures [3]. Low‐intensity electrical currents are known to influence the neuronal excitability, with both pro‐ and anticonvulsant potential. In contrast, low‐frequency electrical currents employed in therapeutic brain stimulation techniques have frequently been used to modulate brain activity in both experimental studies and human patients with severe drug‐resistant refractory epilepsy [4, 5]. In veterinary medicine, however, data on neurostimulation as a preventive measure for epileptic seizures in canine patients remain scarce [6]. This case report describes the successful management of a dog suffering from status epilepticus after being entangled in an agricultural electric fence.

## 2 Case Summary

A 1.5‐year‐old male Kangal Shepherd Dog mix weighing 62 kg presented to the emergency service after being entangled in an agricultural electric fence. The dog was a trained herding dog and was left alone with a livestock herd in an enclosed paddock secured with an electric fence. The electrical fence was connected to a 12‐V battery as a power supply and an energizer to convert the power into a high‐voltage pulse, supplying a brief 10,000‐V impulse every 1.5 s. The owners returned after a few hours to find the dog wholly entangled in the electric fence of the enclosure. The dog's fur was soaking wet from a recent rainfall, and based on the weather conditions, it was estimated that the dog must have been entrapped for approximately 1–2 h before being rescued. The dog was unable to stand and showed stiff limbs, trismus, and hypersalivation when found by the owners.

On presentation to the clinic, the dog was recumbent and unable to stand. It showed severe ptyalism, panting, tachycardia (128/min), and normal body temperature (38.3°C). Because of the dog's large size, recumbency, and limited staff availability during emergency hours, a full neurologic examination was not feasible. However, mentation, cranial nerve function, and responses to noxious stimuli in all four limbs were assessed and found to be normal. Additional physical examination was unremarkable, and no burn injuries were seen on the dog's body.

An ECG and thoracic radiographs were performed to rule out cardiac arrhythmias and noncardiogenic pulmonary edema, respectively. Neither ECG nor radiographs showed abnormalities. Point‐of‐care ultrasound revealed findings suggestive of hypovolemia but no signs of effusion or other pathologies in the thoracic or abdominal cavity. Initial laboratory evaluation consisted of CBC^1^ and venous blood gas analysis^2^. CBC was unremarkable, while blood gas analysis showed severe lactic acidosis (lactate 13.1 mmol/L [118.0 mg/dL]; reference interval [RI]: 1–1.8 mmol/L [9.0–16.2 mg/dL]; pH 7.12, RI: 7.35–7.45; PvCO_2_ 41.8 mm Hg [5.6 kPa], RI: 32–45 mm Hg [4.3–6.0 kPa]; HCO_3_^−^ 13.2 mmol/L [13.2 mEq/L], RI: 20.0–24.0 mmol/L [20.0–24.0 mEq/L]; base excess −15.7 mmol/L [−15.7 mEq/L], RI: −3.0 to 3.0 mmol/L [−3.0 to 3.0 mEq/L]). Because hemostasis can also be affected by electrocution injury, thromboelastography^3^ was performed, which was normal. Troponin I measurement from the time of presentation was performed by an external laboratory^4^ and showed a marginal increase (0.14 ng/mL; RI: <0.13 ng/mL).

Stabilization measures consisting of oxygen supplementation and fluid administration were initiated when the dog suddenly developed generalized tonic‐clonic seizures. Midazolam^5^ (0.5 mg/kg, IV) was administered, with temporary effect, and had to be repeated twice after signs progressed to status epilepticus. Treatment was quickly extended to a constant rate infusion (CRI) of midazolam (0.5 mg/kg/h), and overt seizure activity abated. Approximately 30–60 min later, the dog again experienced refractory seizures, necessitating a stepwise escalation of antiseizure therapy that began with a loading dose of phenobarbital^6^ (16 mg/kg, IV, administered in fractioned doses of 2 mg/kg every 20 min). When seizures persisted, levetiracetam^7^ (60 mg/kg, IV) was added, followed by ketamine^8^ (0.3 mg/kg/h, CRI) and finally dexmedetomidine^9^ (3 µg/kg/h, CRI), at which point overt seizures stopped [7]. Although clinically visible seizure activity had ceased, progressive antiseizure and sedative therapy had led to hypoventilation, as evidenced by a progressive increase in PvCO_2_ on blood gas analyses, and a decrease in respiratory rate. Consequently, mechanical ventilation^10^ was initiated approximately 15 h after initial presentation. The patient was intubated with a 12.0‐mm endotracheal tube, and pressure‐controlled ventilation mode was used. Initial ventilator settings included a peak pressure of 13 cm H_2_O (10 cm H_2_O over positive end‐expiratory pressure [PEEP]), PEEP of 3 cm H_2_O, a respiratory rate of 16/min, and an FiO_2_ of 50%. These settings were adjusted as needed throughout the dog's treatment based on repeated blood gas analyses, clinical monitoring, and the patient's response.

Considering the dog's continued deterioration and financial constraints, the owners briefly considered euthanasia. A 24‐h electroencephalogram (EEG) was performed while the dog was undergoing mechanical ventilation, helping to guide treatment and provide prognostic information for the owners. EEG monitoring was used to assess for the presence of nonconvulsive status epilepticus [8], which can be challenging to detect clinically in the absence of overt signs of seizure, particularly in heavily sedated patients. EEG recording was performed using a commercial EEG device^11^ and both a monopolar and bipolar montage of electrodes. Ten subdermal wire electrodes^12^ were fastened to the scalp with a bandage according to a previously described standard protocol for dogs [9]. Subdermal wire electrodes included frontal electrodes (F3/F4), central electrodes (C3/C4), temporal electrodes (T3/T4), occipital electrodes (O1/O2), and reference (R) and ground (G) electrodes. Impedances of the electrodes were checked regularly and kept below 10 kOhm. In addition, 0.1‐ to 1‐Hz low‐frequency and 35‐ or 70‐Hz high‐frequency filters were applied and adjusted depending on muscle artifacts.

**FIGURE 1:**
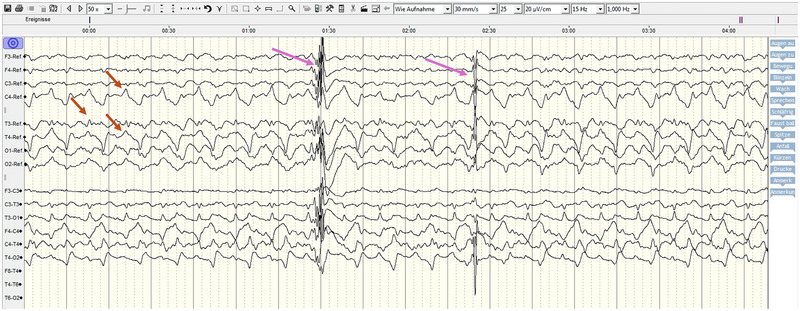
Electroencephalogram (EEG) excerpt from a 24‐h monitoring period in a 1.5‐year‐old Kangal Shepherd Dog mix with status epilepticus after electrocution. The trace demonstrates epileptiform activity characterized by repetitive spikes and sharp waves with high amplitude appearing as bursts and interrupting the background rhythm (long arrows). Additionally, there is an approximately 1.2‐Hz rhythmic slow pattern with sharp‐wave morphology, predominantly over electrodes C4, T4, and T3 (short arrows). These findings were observed during the initial hours of monitoring and are consistent with ongoing seizure activity. The EEG was recorded using a 10‐channel subdermal wire electrode system. Sensitivity: 20 µV/cm; sweep speed: 30 mm/s.

**FIGURE 2:**
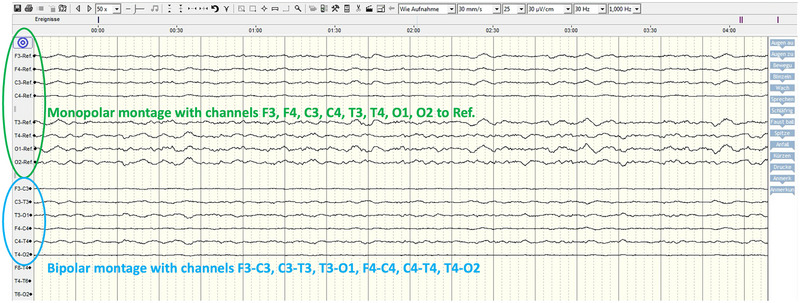
Electroencephalogram (EEG) excerpt from the same 24‐h monitoring period in a 1.5‐year‐old Kangal Shepherd Dog mix after electrocution. This trace shows physiologic background activity composed of high‐voltage, low‐frequency rhythms (predominantly theta and delta activity). No epileptiform discharges were observed during this segment. The EEG was recorded using a 10‐channel subdermal wire electrode system. Sensitivity: 20 µV/cm; sweep speed: 30 mm/s.

Twenty‐four‐hour EEG monitoring was performed continuously and checked hourly throughout the night. EEG background waves corresponded to the various sleep phases, with high‐voltage, low‐frequency rhythm mostly observed. Multiple spikes and sharp waves were observed in the first hours of examination, supportive of ongoing status epilepticus (Figure 1). The seizure activity progressively decreased within this initial period, and physiologic background activity without epileptiform discharges was observed for the remainder of the monitoring period (Figure 2).

Based on the absence of epileptiform discharges on the EEG after 24 h, antiseizure and sedative CRIs were slowly tapered in reverse order of their initiation. The dog was successfully weaned from ventilatory support after a total of 30 h. Antiseizure medication at that stage still consisted of phenobarbital^6^ (3 mg/kg, IV, q 12 h) and levetiracetam^7^ (20 mg/kg, IV, q 8 h).

One day after successful weaning from the ventilator, the dog was still unable to stand, was lethargic, and showed an increase in body temperature (40.3°C). Laboratory testing^13^^,^ 14 revealed increased C‐reactive protein (38.9 mg/L [3.9 mg/dL]; RI: 0–10.8 mg/L [0–1.0 mg/dL]); creatine kinase (9660 U/L; RI: <143 U/L), moderate leukocytosis (21.77 × 10^9^/L [21.77 × 10^3^/µL], RI: 5.48–13.74 × 10^9^/L [5.48–13.74 × 10^3^/µL]), and neutrophilia (15.94 × 10^9^/L [15.94 × 10^3^/µL], RI: 2.78–8.73 × 10^9^/L [2.78–8.73 × 10^3^/µL]). Based on observed progressive tachypnea (52/min), thoracic radiographs were recommended; however, the client declined. Therefore, empiric antimicrobial therapy with amoxicillin–clavulanic acid^15^ (20 mg/kg, IV, q 8 h) was initiated for the treatment of presumed aspiration pneumonia. Five days later, the owners agreed to thoracic radiographs before discharge, which showed no abnormalities.

On Day 2 after weaning from mechanical ventilation, the dog showed slow improvement and was able to stand with support but not yet able to walk. Antiseizure medication was gradually tapered over the following days, transitioning from IV to oral administration of phenobarbital and progressively reducing IV levetiracetam until it was discontinued, leaving only oral phenobarbital. Additionally, physiotherapy was initiated to support the recovery process. Four days after weaning from mechanical ventilation, the dog was able to walk with support. Two days later, the dog's clinical status further improved, and it was able to stand and walk independently. The dog was discharged after a total of 7 days. Blood parameters before discharge indicated a reduction in inflammatory markers and creatine kinase (WBCs 17.55 × 10^9^/L [17.55 × 10^3^/µL], RI: 5.48–13.74 × 10^9^/L [5.48–13.74 × 10^3^/µL]; neutrophils 12.98 × 10^9^/L [12.98 × 10^3^/µL], RI: 2.78–8.73 × 10^9^/L [2.78–8.73 × 10^3^/µL]; C‐reactive protein 27.1 mg/L [2.7 mg/dL], RI: 0–10.8 mg/L [0–1.0 mg/dL]; creatine kinase 795 U/L, RI: <143 U/L).

The dog was discharged with oral phenobarbital^16^ (2.7 mg/kg, q 12 h) and made a full neurologic and clinical recovery. Oral medication was slowly reduced and eventually discontinued. However, 4 weeks after phenobarbital was discontinued, the dog experienced a seizure episode. Because the owners did not want to present the dog for further examination, treatment with oral phenobarbital (2.7 mg/kg, q 12 h) was reinitiated. A follow‐up with the referring veterinary practice found that the dog had been euthanized 2 months after discharge because of recurrent and progressive seizure activity.

## 3 Discussion

The current case describes the rare incident of electrocution injury in a dog after accidental entanglement in an agricultural fence. Electrocution injuries are generally rare in small animals and most commonly result from animals chewing electrical cords. The resulting sequelae of electrocution injury are variable and difficult to predict, as they depend on several factors, such as the voltage, the type of current (AC or DC) and the duration of flow, the path of the electrical current through the body, and the resistance of the tissues within the pathway [10]. Commonly reported injuries in people and animals include cardiac arrhythmias, such as ventricular fibrillation, pulmonary edema, and burn injuries [1, 11]. Neurologic signs, such as seizures and spinal cord damage, have also been reported in people [12] and in one dog after it chewed a power cord, resulting in electrocution [13].

The exact pathophysiology behind electrocution injuries is still not fully understood, but several mechanisms have been identified or hypothesized. Direct and indirect damage can occur with the transformation of electrical energy into thermal energy, resulting in superficial or internal tissue damage caused by heat generation. Additionally, electricity can directly affect cardiac cell conduction and cause arrhythmias [1]. Neurologic sequelae are likely associated with direct physiologic damage to the cells caused by electricity. Electric shock can cause electroporation, a process that creates pores in the cell membranes and leads to macromolecule leakage and secondary osmotic swelling, edema, and cell necrosis [10, 12]. Additionally, demyelination resulting from electrical injury and indirect nerve damage associated with nerve compression due to edema formation in the surrounding tissue can occur [12].

An electrical current preferentially travels along the shortest path with the least resistance to a grounding source. Bones, tendons, fat, and skin have the highest resistance in the body. In contrast, blood, muscle, and nervous tissue have the lowest resistance, making these tissues more susceptible to damage from electrical trauma. Additionally, while an electrical current must overcome a resistance of approximately 100 kOhm with dry human skin, this resistance can drop to approximately 1 kOhm if the skin is wet [10]. Fur and wool can further increase resistance, which is likely why the manufacturers of agricultural fences recommend higher voltages for effective confinement of large farm animals [1]. Damage from electrical current traveling through low‐resistance tissues, such as blood vessels or neurons, can result in thrombosis, edema, ischemia, and alterations of cell membranes, ultimately leading to neurologic signs [10, 12].

The kindling phenomenon may represent a potential pathophysiologic mechanism in the current case. Kindling is a process by which repeated subthreshold electrical or chemical stimulation of specific brain regions, particularly within the limbic system, can lead to progressively more intense and, eventually, spontaneous seizures [14, 15]. Although originally described in experimental models, kindling is considered a contributing factor in the development of epilepsy in both people and animals [14, 16]. It is plausible that repeated electrical stimulation during the dog's prolonged entrapment in the electric fence acted as a stimulus, ultimately contributing to the development of status epilepticus and recurrent seizure activity. This mechanism may partly explain the delayed seizure recurrence after initial recovery, emphasizing the potential for chronic epileptogenic consequences in animals that survive electrocution injuries.

The current case is the first reported incident of a dog presenting with status epilepticus after electrocution. The differences in voltage, current, and pulse duration from the electric fence likely played a role in the dog's clinical presentation with seizures rather than the classic cardiorespiratory signs seen with electric cord–related electrocution.

Retrospectively, it is difficult to assess exactly which factors contributed to the underlying mechanisms at hand, including the dog's wet fur, the multiple contact points of the electrical strands, and the large grounded surface area from lateral recumbency, all of which might have contributed to the extent of the dog's injuries and clinical manifestations. Thus, the extent and clinical presentation associated with electrocution injuries are difficult to predict and vary among individual cases.

Although the dog had no known previous health concerns and the owners suggested the entanglement likely occurred during an attempt to breach the fence, it cannot be fully excluded that seizure activity may have preceded the entanglement and contributed to the incident.

Only a few documented reports regarding electrocution injury in small animals currently exist, mostly with unfavorable outcomes [13, 17, 18]. One important factor that might have affected the short‐term outcome in the current case was the decision to perform an EEG. EEGs have been used in people and animals to guide anesthesia depth and assess seizure control in status epilepticus patients [19, 20]. Although clinically detectable seizures might cease with antiseizure therapy, it is possible the patient still experiences seizure activity only notable on EEG. Thus, the aim of EEG monitoring is to evaluate electrical brain activity and provide information on otherwise undetectable, nonconvulsive seizure activity through the interpretation of different wave patterns and detection of abnormal epileptiform waveforms. EEG was used in the current case to help detect nonconvulsive seizures and determine when medication should be tapered and a new weaning attempt from mechanical ventilation should be conducted. The background activity observed in Figure 1 (short arrows) demonstrates an approximately 1.2‐Hz rhythmic slow pattern with sharp‐wave morphology, predominantly over electrodes C4, T4, and T3. This pattern may be suggestive of periodic epileptiform discharges such as periodic synchronous discharges or periodic lateralized epileptiform discharges, which are commonly associated with nonconvulsive status epilepticus. Although this interpretation is plausible, it cannot be definitively confirmed due to the absence of a simultaneously recorded ECG trace during EEG acquisition, which precludes the exclusion of potential cardiac artifacts contributing to the observed rhythmicity. Nonetheless, these findings warrant consideration as possible manifestations of nonconvulsive seizure activity.

Long‐term EEG recording showed physiologic background activity with absent epileptiform discharges, which was consistent with the clinical improvement observed after escalation of antiseizure therapy. This finding supported the impression that seizure control had been achieved and provided an initial short‐term, positive prognostic indicator for neurologic recovery. Monitoring the brain's electrical activity also supported the owners’ decision to continue treatment, because no severe abnormalities were observed within the 24‐h EEG monitoring.

Despite clinical improvement and a seemingly full recovery initially, the dog again experienced seizures after discontinuation of the antiseizure medication and was ultimately euthanized several months later for progressive seizures. In people, long‐term neurologic effects after electrical injury, such as epilepsy, migraines, vertigo, and blindness, have been reported to either persist chronically or develop as delayed sequelae, even months or years after the initial electrical injury [12, 21]. The recurring seizures in the current case might have been long‐term effects of the electrical trauma. In hindsight, continued or long‐term antiseizure therapy might have been advisable given the severity of the initial presentation and the risk of chronic epileptogenicity. A key challenge in such cases is the limited knowledge and scarce veterinary literature regarding long‐term neurologic consequences of electrical injury in dogs, which complicated the decision‐making regarding prognosis and duration of treatment. This case emphasizes the importance of long‐term neurologic monitoring and cautious decision‐making in similar cases when considering discontinuation of antiseizure therapy. Further diagnostic tests, including magnetic resonance imaging, would have been desirable to assess the full extent of the neurologic damage caused by electrocution and would have proven valuable for future cases of this rather rare but serious type of injury.

To the authors’ knowledge, this case report is the first to describe the successful management, including mechanical ventilation and EEG monitoring, of a dog with status epilepticus associated with electrocution injury from an electric fence. The case highlights the potential dangers of electric fences, which are commonly considered harmless, particularly if animals become entangled. EEG monitoring provided valuable therapeutic and prognostic insights and played a crucial role in the positive short‐term clinical outcome of this case. Furthermore, the current case raises awareness that despite the initial apparent clinical recovery, dogs surviving electrocution injuries may remain susceptible to the long‐term neurologic sequelae similar to those described in people.

## Ethics Statement

The authors confirm that the ethical policies of the journal, as noted on the journal's author guidelines page, have been adhered to. No ethical approval was required as this is a case report with no original research data.

## Conflicts of Interest

The authors declare no conflicts of interest.

## Endnotes

1 ProCyte Dx, IDEXX Laboratories, Westbrook, ME.

2 Cobas b 123 System, Roche Diagnostics Deutschland GmbH, Mannheim, Germany.

3 TEG 5000, Haemonetics Corp., Braintree, MA.

4 Troponin I (Chemiluminescence Immuno Assay), Biocontrol, Ingelheim, Germany.

5 Midazolam‐hameln, Hameln Pharma GmbH, Hameln, Germany.

6 Luminal, Phenobarbital‐Na, Desitin Arzneimittel GmbH, Hamburg, Germany.

7 Levetiracetam Hikma, Hikma Pharma GmbH, Martinsried, Germany.

8 Ketamin, Serumwerk Bernburg AG, Bernburg, Germany.

9 Dexdomitor, Dexmedetomidinhydrochlorid, Orion Corporation Orion Pharma, Espoo, FL.

10 Elisa 800 Ventilator, Löwenstein Medical SE & Co. KG, Bad Ems, Germany.

11 Neurofax EEG, Nihon Kohdin Co. Ltd. Tokyo, Japan.

12 Subdermal wire electrodes, IVES EEG Solutions Inc, Newburyport, MA.

13 ADVIA 2120, Siemens Medical Solution Diagnostics, Eschborn, Germany.

14 DxC 700 AU Chemistry Analyzer, Beckman Coulter, Brea, CA.

15 AmoxClav Hexal, Hexal AG, Holzkirchen, Germany.

16 Phenoleptil, Phenobarbital, Dechra Regulatory B.V., Bladel, Netherlands.
